# Attachment style, ways of coping with stress and life attitudes by MSM who are active chemsex users

**DOI:** 10.1192/j.eurpsy.2022.2074

**Published:** 2022-09-01

**Authors:** P. Kaluzny, R. Kowalczyk

**Affiliations:** Andrzej Frycz Modrzewski Krakow University, Psychology, Krakow, Poland

**Keywords:** stigma, drugs, chemsex

## Abstract

**Introduction:**

The research was carried out in Poland between 7-19 March 2021 with the help of online questionnaire on a group of 183 male aged 18-20. The subjects were divided into two age groups: 18-29 and 30-40. The second division are chemsex and non chemsex users.

**Objectives:**

Objective of this research was to test whether attachment styles, ways of coping with stress and feeling of sense of life influence the risk of overusing chemsex in MSM groups aged 18-29 and 30-40.

**Methods:**

Online questionnaire composed of demographics and 3 psychological tests: Ways of Attachment Questionnaire (Polpa 2008) Ways of Coping Questionaire (polish adaptation by P. Szczepaniak, J. Strelau, K. Wrześniewski) Life Attitude Profile - Revised (polish adaptation by R. Klamut)

**Results:**

Examinated chemsex users, based on life attitudes analysis, tend to lead a strongly oriented life, are convinced of having clear and well-defined goals. In terms of dealing with stress they are presenting focused-on-task style, they exhibit lowered levels of avoidant style than normally characteristic for addicts. The research did not reveal any connection between chemsex users and attachment styles.

**Conclusions:**

The research did not allow to clearly point out any connections between risk factors and chemsex usage. During data analysis some weak links occurred, nevertheless too weak to state any risk factors. As results of carried out research it was possible to determine some models, which marked some specific values, obtained on given scales, and following connections between heightening and lowering chance of chemsex usage.

**Disclosure:**

No significant relationships.

